# “It changed the atmosphere, but how long will it last?”: healthcare professionals’ experiences of an intervention to improve positive communication in intensive care

**DOI:** 10.1016/j.aicoj.2026.100047

**Published:** 2026-03-19

**Authors:** Anita Barth, Maurizio Cecconi, Carole Boulanger, Elie Azoulay, Nancy Kentish Barnes

**Affiliations:** a European Society of Intensive Care Medicine (ESICM), Brussels, Belgium; b Department of Nursing and Integrative Health Sciences, University of Debrecen, Debrecen, Hungary; c Biomedical Sciences Department, Humanitas University, Pieve Emanuele, Italy; d Department of Anaesthesia and Intensive Care, IRCCS Humanitas Research Hospital, Rozzano, Milan, Italy; e Royal Devon University NHS Foundation Trust, Barrack Road, Exeter, UK; f Medical Intensive Care Unit Department, AP-HP, Saint-Louis University Hospital, Paris-Cité University, Paris, France

**Keywords:** Mental health, Burnout, Shortage, Positive communication, Intensive care

## Abstract

**Background:**

Burnout among healthcare professionals (HCPs) working in intensive care units (ICUs) has reached alarming levels, with detrimental effects on staff well-being, patient care, and organizational functioning. The HELLO randomized controlled trial (RCT) tested a multicomponent intervention aimed at reducing burnout by promoting positive communication and teamwork. This qualitative study complemented the RCT by exploring participants’ experiences and perceptions of the intervention, identifying barriers, facilitators, and factors influencing sustainability.

**Methods:**

This qualitative component was conducted alongside the HELLO cluster-RCT in ICUs across multiple countries. Data sources included (1) 1,155 pictures of messages placed in “HELLO” boxes and on noticeboards, and (2) 26 semi-structured interviews with 27 ICU professionals from 18 ICUs, conducted 4–5 months after the intervention. Data were analysed using thematic analysis.

**Results:**

Box messages from 26 ICUs in 20 countries revealed recurrent themes of appreciation, recognition, kindness, teamwork, and motivation, often expressed through short, personal notes of gratitude or encouragement. Interviews identified six overarching themes: understanding of the intervention and initial reactions; facilitators and barriers to implementation; focus on messages; local adaptations; immediate positive outcomes; and long-term potential outcomes. Participants reported improved team cohesion, communication, and workplace atmosphere, with leadership engagement emerging as a key facilitator. Barriers included scepticism, workload, and pre-existing workplace tensions. Although many perceived short-term benefits, most noted that the effects diminished over time unless reinforced through continued initiatives.

**Conclusions:**

This qualitative study provides insight into healthcare professionals’ experiences of the HELLO intervention, highlighting perceived improvements in communication and team atmosphere, as well as challenges related to engagement, workload, and sustainability. These findings can inform future adaptations and implementation strategies for similar low-cost interventions in intensive care units.

**Trial registration:**

ClinicalTrials.gov NCT06453616 (June 18, 2024).

## Introduction

Burnout has increased over the past decade among healthcare professionals (HCPs) [1], and it remains a major concern especially among those working in intensive care units (ICUs) [2,3]. Burnout, defined as a syndrome of emotional exhaustion, depersonalization, and a diminished sense personal accomplishment [4], has serious consequences for staff well-being, patient outcomes, and the overall functioning of the healthcare system [5].

Numerous strategies have been proposed to prevent or reduce burnout [6,7]. Evidence suggest that the most effective approaches address both organisational and individual aspects. However, many existing programmes still focus primarily on individuals, while organizational components are often overlooked [[8], [9], [10], [11]]. Nevertheless, a recent systematic review highlighted that many burnout interventions still lack rigorous evaluation, particularly with regard to their long-term effectiveness and sustainability [12]. This underscores the need for comprehensive, rigorously assessed strategies that combine different methods to create healthier workplace environments [13,14].

The primary aim of the HELLO randomized controlled trial (RCT) was to test a multicomponent intervention designed to reduce burnout among HCPs in ICUs, with evaluation of burnout prevalence as its main outcome [15]. Results from the RCT indicated that the intervention was associated with a reduced prevalence of burnout 4 weeks after implementation. However, trial outcomes alone cannot capture the mechanisms underlying these effects or the contextual factors influencing uptake and sustainability. A qualitative approach therefore complements the RCT by providing in-depth insight into HCPs’ experiences, perspectives, and subjective responses, while also identifying barriers and facilitators to implementation. Such understanding is essential for interpreting effectiveness and assessing the long-term viability of the intervention. Accordingly, in this qualitative study, we sought to explore how HCPs experienced the intervention, including its perceived benefits, challenges, and factors influencing implementation and sustainability.

## Methods

### Study design and setting

This qualitative component was part of a larger multicentre, cluster-randomized controlled trial that aimed to reduce burnout and improve relationships and teamwork among healthcare professionals (HCPs) in intensive care units (ICUs) [16]. During the trial period a four-week intervention was implemented as described in the published study protocol. The intervention consisted of 6 components: posters, email reminders, greetings during morning meetings, role modelling, and positive messages placed in boxes and on noticeboards. Each component was designed to promote positive social interactions and a supportive work environment. The posters served as visual cues to reinforce desired behaviours and social norms. Email reminders were sent twice a week to the local investigators, who then shared them with their team members to encourage continued engagement with the intervention. Greetings were included into morning meetings to help build team cohesion and set a positive tone for the day. Nursing and medical leaders acted as role models, setting an example for others to follow. Additionally, staff members could leave positive messages for their colleagues in the boxes and on noticeboards, boosting morale, fostering a supportive work environment and a sense of community [15]. In addition, a video explaining the intervention was made available to the ICUs in the intervention arm (https://vimeo.com/988364914/e2c89b435b).

**Fig. 1:**
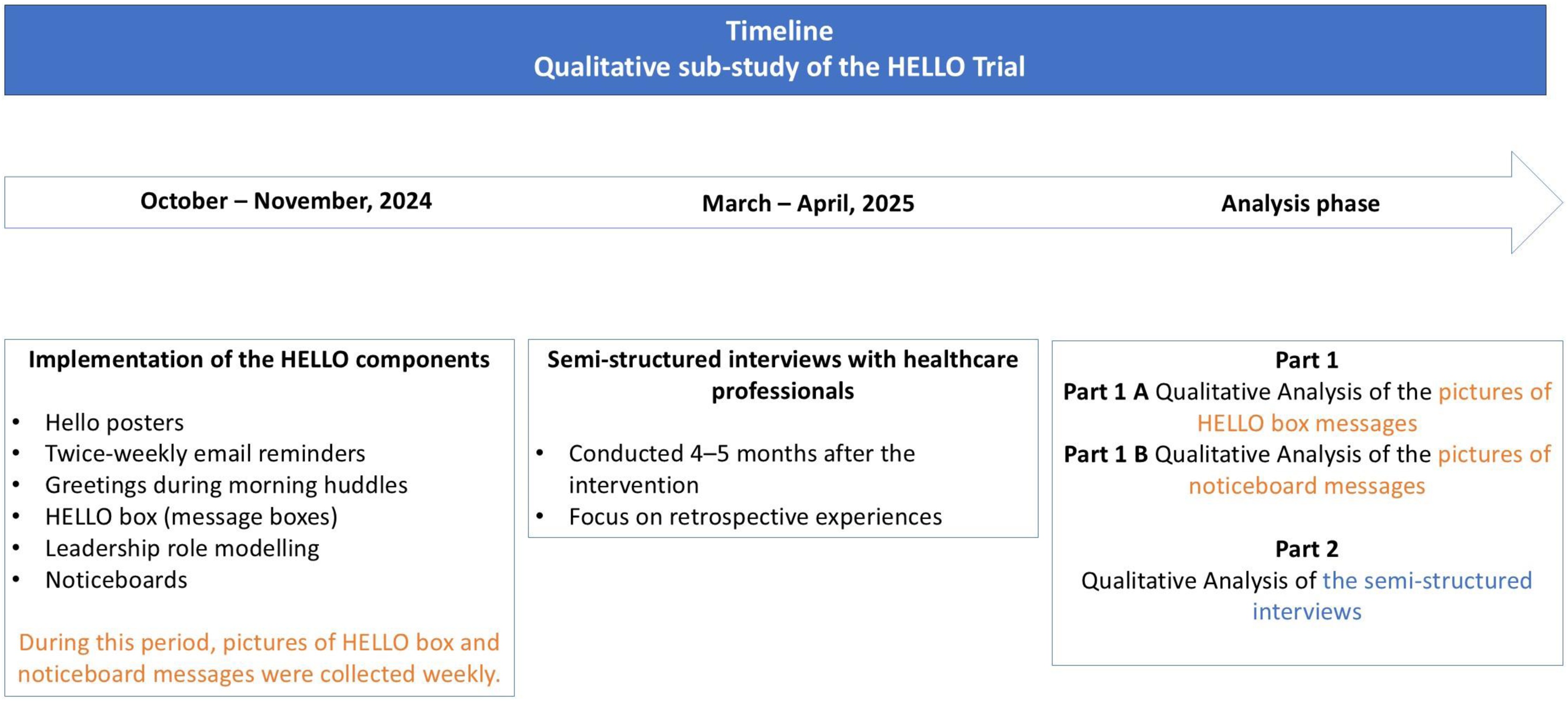
Timeline of the HELLO intervention and qualitative sub-study.

To better understand how the intervention was implemented as well as HCPs’ experiences, the qualitative component consisted of two parts. First, pictures of messages placed in boxes and on noticeboards during the intervention were collected and analysed. Second, semi-structured interviews were conducted with HCPs who had direct experience with the intervention to explore their experiences and perceptions of its implementation and impact (Fig. 1).

Participating ICUs applied for institutional review board (IRB) approval according to legislation in the relevant country s specified in the study protocol [16]. The trial was registered on ClinicalTrials.gov on June 18, 2024 (NCT06453616).

### Data collection

Data were collected through two sources: pictures of the written messages and semi-structured interviews.

First, local investigators at each intervention ICU were asked to send pictures of the messages placed in boxes and on noticeboards weekly throughout the four-week intervention period for further analysis. The pictures can be accessed online via the following link: https://www.dropbox.com/scl/fo/qt8z4w3zk1ako7xuganx4/AED_EQk6vuwrxGSp3eMLmvM?rlkey=ispyrsenwlwlpv5xkkr5r8ewh&e=1&st=7ak60m04&dl=0.

Second, semi-structured interviews were conducted with HCPs actively engaged in the intervention between March and April 2025, 4–5 months after the intervention ended. Investigators were asked to identify staff members, ideally one medical doctor and one nurse, who were willing to participate in the interviews. All 370 centres included in the final analysis of the HELLO trial were contacted. Of these, 39 centres initially expressed interest, but only 18 subsequently responded and scheduled an interview appointment. After participants were identified, local investigators facilitated communication between the interviewees and the interviewers. All interviews were held virtually through Microsoft Teams. Interviews were conducted by 2 researchers, in either English or French, depending on participant preference. An interview guide was developed and used to explore participants’ experiences, perceptions of the intervention’s impact, and suggestions for improvement. Demographic data were self-reported and included age, sex, role in intensive care unit, and years of experience working in intensive care. Interviews were audio-recorded with participant consent and transcribed verbatim.

### Data analysis

Data from both the collected pictures and the interview transcripts were analysed using thematic analysis between May and July, 2025.

**Fig. 2:**
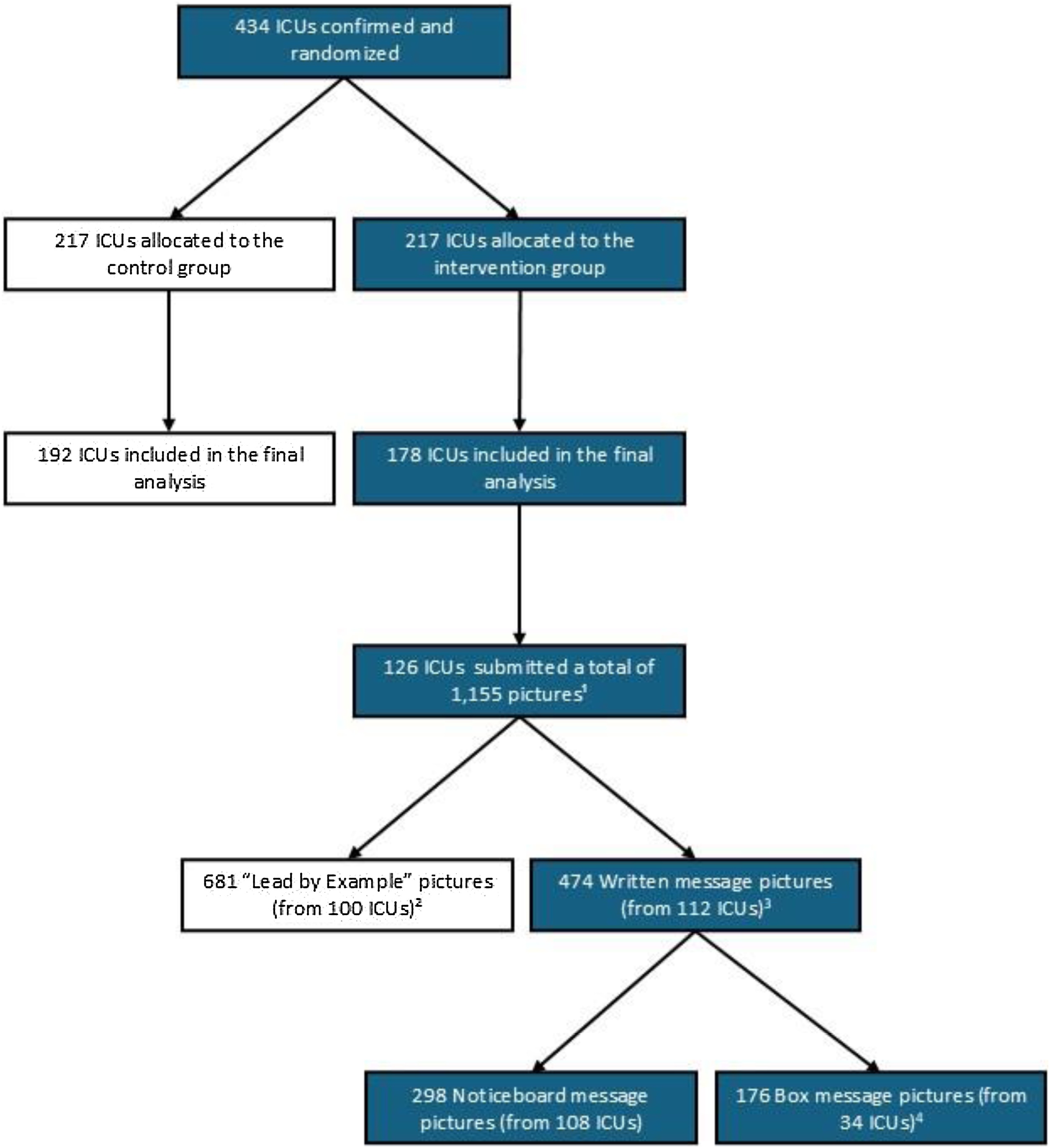
Written messages from the box and noticeboard. Note: 1. Some ICUs sent multiple types of pictures (e.g. Team involvement, box & noticeboard messages), while others sent only one or two types. 2. The pictures showed for example the Hello poster, the box and noticeboard in place and ready to be used, team members posing as a team, staff members writing messages on the noticeboard. 3. Included in the qualitative analysis. 4. Several pictures contained more than one message, which was individually coded during analysis.

The analysis of the box and noticeboard messages, started with carefully checking and categorising the pictures received from the local investigators to identify those containing messages suitable for analysis, as many included group photos or pictures without message content. 73% of all ICUs in the intervention group who were included in the final quantitative analysis sent pictures. A total of 1,155 pictures were received, 474 of which showed written messages from the box or noticeboard in multiple languages (Fig. 2).

In the first phase, box messages were analysed using the following steps:

1 **Transcription**: All messages visible in the pictures of the box messages were transcribed, with the support of ChatGPT in the process. Each transcription was carefully double-checked.
2 **Translation**: Translation into English was carried out by members of the research team who were either native speakers of the respective languages or had good working proficiency. For languages not spoken by members of the research team, online translation tools (DeepL, ChatGPT) were used. Since the messages were generally short and simple the translation was straightforward.
3 **Coding**: Inductive coding was used to identify key domains emerging from the box messages.
4 **Codebook development**: A codebook was developed by two researchers, based on the initial coding.
5 **Data saturation**: Coding continued until no new codes emerged. Data saturation was reached after coding 296 box messages from 26 ICUs.
6 **Finalizing themes**: Themes were defined and named.

In the second phase, the noticeboard messages were analysed using the following steps:

1 **Transcription:** All messages in the pictures of the noticeboard messages were checked, however due to the large number of messages only a selection of examples were transcribed to represent the codes. ChatGPT was used to assist with the transcription process. Each transcription was carefully double-checked.
2 **Translation**: Followed the same process as for the box messages.
3 **Coding**: The previously developed codebook was used to code the noticeboard messages. When messages did not fit the existing codes, new codes were created accordingly.
4 **Data saturation**: Coding continued until no new codes emerged. Data saturation was achieved after coding noticeboard messages from 21 ICUs.
5 **Finalizing themes**: Followed the same process as for the box messages.

The analysis of the interview transcripts followed the structured process described below:

1 **Initial thematic review**: two researchers independently coded two randomly selected interview transcripts and identified key themes and concepts that occurred in the interviews.
2 **Codebook development**: A codebook was developed through a systematic comparison of the initial codes generated by each researcher and continued until consensus was reached.
3 **Transcript coding**: The codebook developed was used to code the remaining interview transcripts and was further adjusted as needed to accommodate new codes.
4 **Data saturation**: Coding continued until no new codes emerged. Data saturation was achieved after 23 interviews had been conducted. However, three additional interviews were carried out, as they had already been scheduled with the participants.
5 **Finalizing themes and quotes**: Themes and sub-themes were defined and named. Representative quotes were selected to illustrate each sub-theme and support the interpretation of findings (Table 2).

While both datasets were analysed thematically, they were interpreted with different analytical aims: intervention materials were examined to understand how teams appropriated the proposed tools, whereas interviews were analysed to explore participants’ retrospective experiences of the intervention.

## Results

### Findings from box and noticeboard messages

**Fig. 3:**
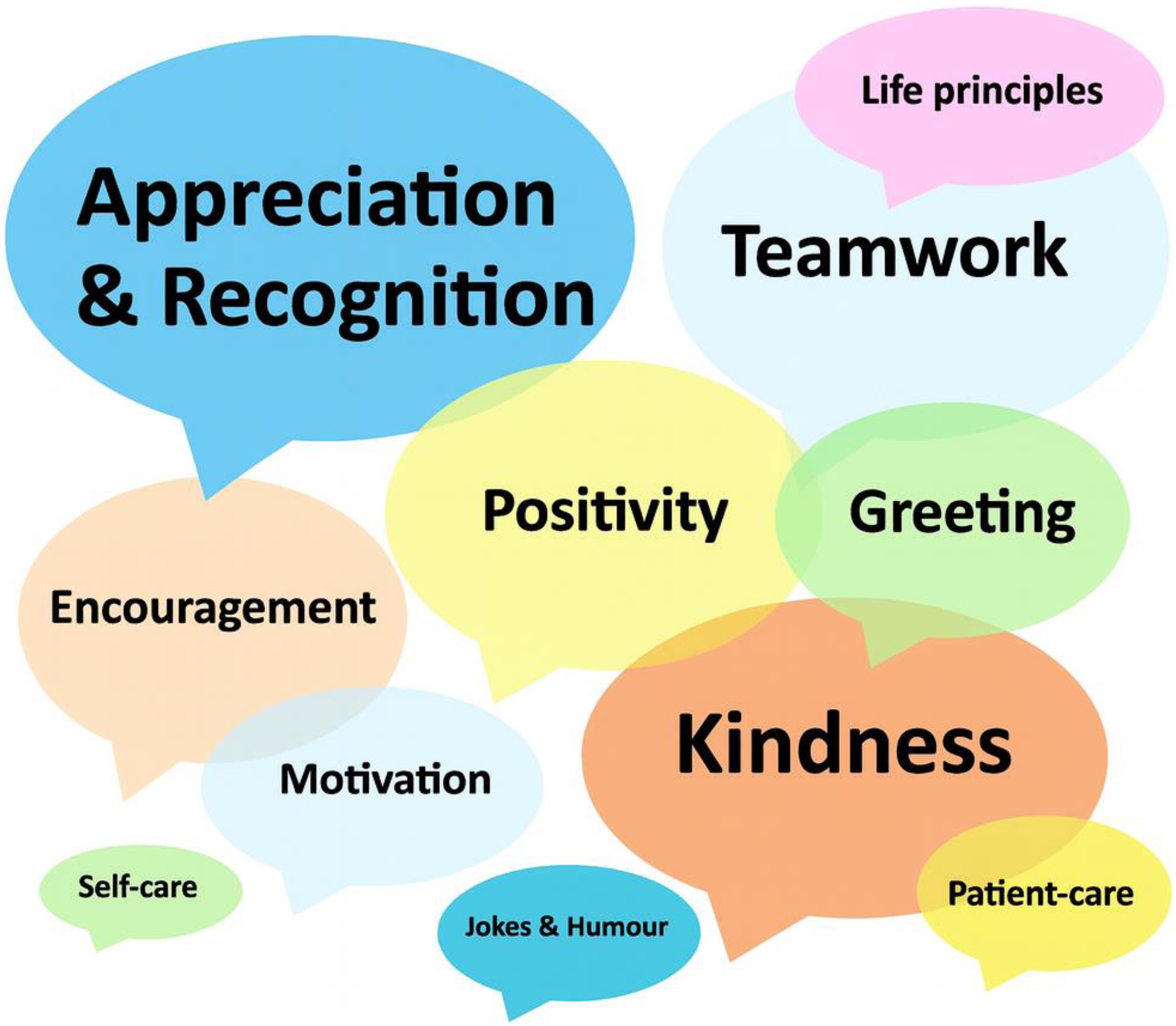
Key themes from box message analysis. Note: Larger bubbles indicate themes that appeared more frequently across box messages.

Box messages were analysed from 26 ICUs across 20 countries. The messages were written in 8 languages: Dutch, English, French, German, Italian, Portuguese, Romanian, and Spanish. Most messages expressed appreciation, recognition, and gratitude for colleagues and the team, often naming staff members and acknowledging their contributions. Personal notes were identified in messages from 11 ICUs, with the highest number coming from the United States and France. Numerous messages reflected kindness, teamwork, and positivity *(“Our greatest strength is unity. Together we are better, as people and professionals, and happier”*). Encouragement and warm greetings were also common, alongside motivational remarks *(“Good morning ICU Team, I must commend the entire team for your dedication and passion towards these patients. It's cool working with you”*). In addition, messages related to the importance of patient care and self-care were also present. Finally, a few messages included humorous notes and jokes. A word cloud was created to visually represent the 11 key themes, where bigger words indicate greater representation (Fig. 3).

Noticeboard messages were analysed from 21 ICUs across 14 countries. They were similar in content to the box messages, covering themes such as appreciation and recognition, encouragement, humour and jokes, kindness, motivation, patient care, positivity, self-care, teamwork, and greetings. However, they were significantly less personal, with less messages naming staff members. A notable difference was the presence of messages with spiritual or religious content (“*God loves you*” etc.). In addition, many noticeboards included detailed visual elements such as hand-drawn illustrations and symbols. These were mainly seen in ICUs located in China, India, and Taiwan. In some cases, noticeboards were also decorated with small treats like candies and chocolates.

### Findings from semi-structured interviews

**Fig. 4:**
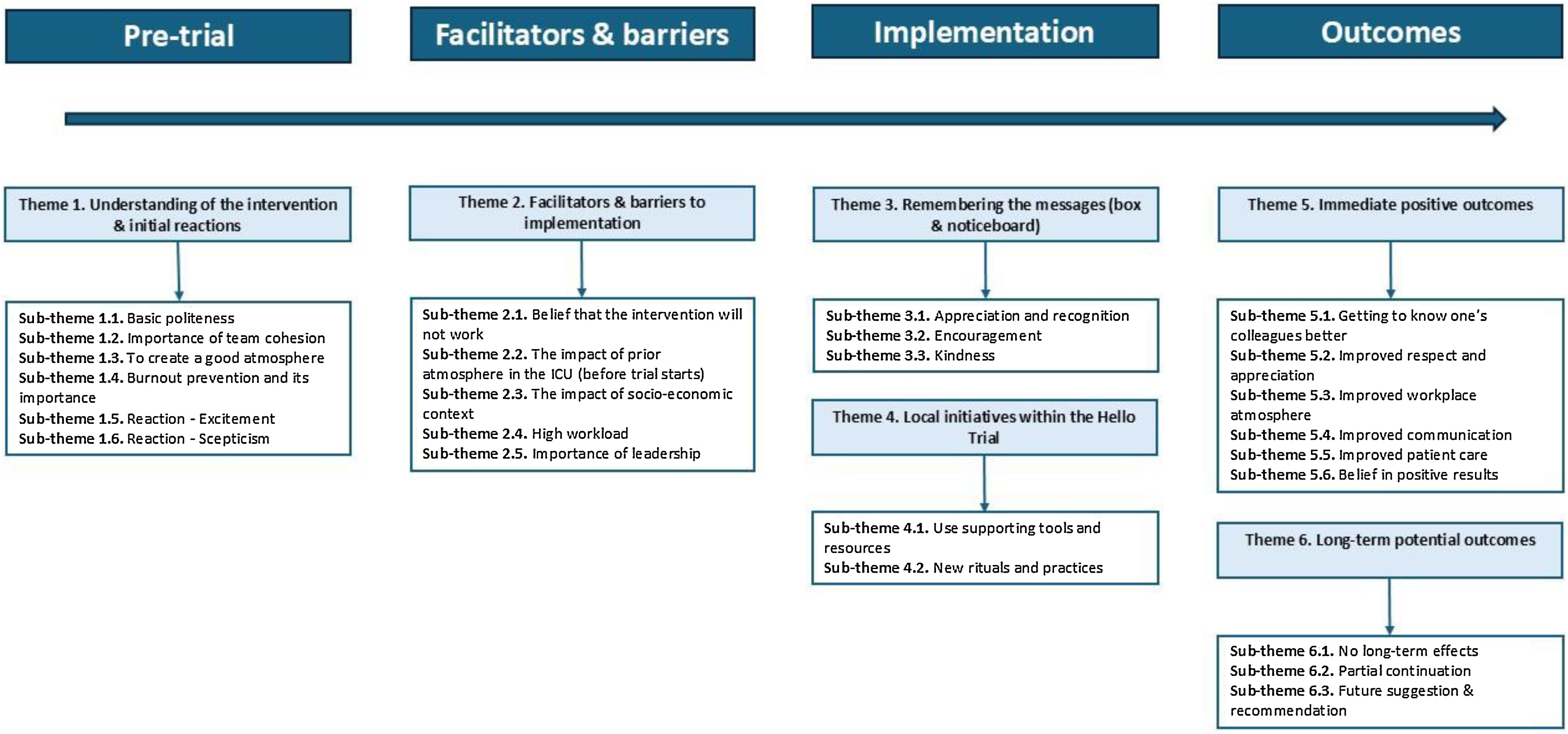
Interview themes and sub-them.

In the 18 ICUs that agreed to participate, a total of 26 interviews were conducted with 27 healthcare professionals, as one interview included two participants. Interviews lasted approximately 20−60 min. The characteristics of the participants are summarized in Table 1. The majority were female (n = 19, 70%). The median age was 43 years, ranging from 26 to 61 years. Most participants were medical doctors (n = 16, 59%). Participants had a median of 13.5 years of experience in intensive care, with a range from 3 to 29 years. They represented a total of 15 different countries from Africa, Asia, Middle East, North, South and Central America, Northern, Southern and Western Europe. The analysis identified 6 major themes, each comprising between 2–6 sub-themes (Fig. 4). A selection of representative quotes illustrating each theme is presented in Table 2.

**Table 1:** Characteristics of interview participants.

Interview number	Country	Age	Sex	Role in intensive care unit	Years of experience in intensive care
1	Colombia	39	Male	Medical doctor	6
2	Colombia	31	Female	Nurse	7
3	Denmark	56	Female	Nurse	25
4	Denmark	61	Male	Medical doctor	25
5	Egypt	44	Female	Medical doctor	16
6	France	50	Female	Nurse	17
7 *	France	42	Female	Medical doctor	15
7 *	France	47	Female	Clinical research unit manager	20
8	France	34	Female	Nurse	3
9	India	47	Female	Medical doctor	21
10	India	47	Female	Nurse	6
11	Italy	30	Male	Nurse	5
12	Italy	37	Male	Medical doctor	5
13	Libya	30	Female	Medical doctor	3
14	Lithuania	26	Female	Nurse	3
15	Lithuania	33	Female	Medical doctor	3
16	Nicaragua	48	Female	Medical doctor	16
17	Nigeria	36	Female	Medical doctor	15
18	Saudi Arabia	57	Male	Medical doctor	26
19	Slovenia	42	Female	Medical doctor	10
20	Spain	50	Male	Medical doctor	20
21	Spain	43	Female	Nurse	12
22	Spain	32	Male	Medical doctor	4
23	Spain	59	Female	Medical doctor	29
24	Spain	48	Female	Nurse	16
25	Taiwan	31	Female	Nurse	7
26	United States	54	Male	Medical doctor	22

* Two participants took part in interview number 7.

**Table 2:** Representative quotes for themes & sub-themes.

Representative quotes for Theme 1. Understanding of the intervention & initial reactions	
Interview number	Sub-theme
	**1.1. Basic politeness**
19	“We should be even more friendly with each other. Not just to avoid unpleasant situations, but especially to be able to talk openly about potential conflicts and other issues.”
21	“I understand that the HELLO trial serves as a reminder of such basic things as arriving on the unit and saying hello, good morning, or goodbye. Something so essential that we already learn in primary school.”
	**1.2. Importance of team cohesion**
14	“I understood it as an intervention in our department aimed at improving connections with our colleagues and overall teamwork.”
26	“When the Hello Trial was introduced, it created an opportunity to bring everyone together, as one team, in one place, and gave us a shared identity, a sense that we all belong to this ICU.”
	**1.3. To create a good atmosphere**
18	“It was more or less about improving communication across the whole ICU team (physicians, nurses, our team, and physical therapists) to create a friendlier environment.”
22	“Well, I understand that the climate in the ICU is more important than we might think or than we used to think. And I believe this intervention will be beneficial for everyone on the team.”
	**1.4. Burnout prevention and its importance**
1	“I think the HELLO Trial is a good initiative from the European Society. It showed the world that this trial is important and that burnout is a serious issue.”
11	“Yes, I read a bit about the Hello Trial, and my boss also explained that the purpose of this trial is to introduce activities aimed at reducing burnout and creating a more comfortable work environment.”
	**1.5. Reaction - Excitement**
4	“I was very surprised how enthusiastic the staff was about this.”
25	“When we invited the physicians or pharmacists and explained that there was a trial aimed at making a positive difference, sharing good things with others, I was surprised by how willing they were to join. They were happy to take part, without any hesitation.”
	**1.6. Reaction - Scepticism**
8	“I'm one of those people who often complains that there's not enough recognition or appreciation between the different professions in our department. So, if that can be improved, I'm all for it. But in terms of the format, focusing on things like saying 'hello,' 'goodbye,' 'thank you,' and 'please', at first, I thought it was a bit ridiculous. These are the most basic expressions of politeness. But then I noticed the difference between when we were part of the study and after it ended, and that really changed my perspective.”
23	“It was very hard to people to understand the purpose of the intervention. They think that we already have a good environment. And they don't think that we need to change anything.”

Representative Quotes for Theme 2. Facilitators and barriers to implementation	
Interview number	Sub-theme
	**2.1. Belief that the intervention will not work**
5	“When I started the study and start enrolling participants, some of them refused because they believed it wouldn’t lead to any real change. I did my best to convince them.”
23	“The main barrier was that some colleagues were not engaged in the process or did not believe in the purpose of the intervention.”
	**2.2. The impact of prior atmosphere in the ICU (before trial starts)**
6	“It happened during a time of tension in the department. There were issues with scheduling and other challenges, so the whole experience felt like a bit of a rollercoaster.”
8	“The fact that suddenly everyone was saying hello to each other felt like too much for me, it didn’t feel natural or spontaneous.”
	**2.3. The impact of socio-economic context**
17	“I believe it’s very important that we also take into account some economic realities. The causes of their stress cannot be resolved with a simple hello.”
25	“I think in traditional Asian culture, people don’t usually speak openly about their feelings. You’re expected to keep it to yourself, because there’s a worry that expressing those feelings might make your colleagues uncomfortable.”
	**2.4. High workload**
13	“Yes, but it didn’t really work. You see, in our ICU, there’s always a staffing shortage, so trying to gather everyone for a morning meeting just wasn’t feasible. I tried, even asked my doctors and senior staff to support it, but it still didn’t work out.”
20	“I have to say that, out of the three locations, the unit in the other building which is more isolated and smaller was the most engaged. It’s a four-bed unit with just two nurses and one physician, and they seemed more enthusiastic and happier to participate. I think they felt more involved and valued, especially since they were taking part in something they’d never experienced before. In contrast, in the larger hospital, where staff are more used to research and various interventions, that same sense of novelty or importance wasn’t really there. Interestingly, even though there were fewer professionals in the smaller unit, the message activity per person was higher. They also had more time, since the patients are less complex, it’s more of an intermediate care unit, which probably helped. Plus, the team there is younger, which might have contributed to their openness and engagement.”
	**2.5. Importance of leadership**
3	“I think the most important thing is that the leaders of the ward set an example by greeting and acknowledging others, because that can spread throughout the ward.”
5	“And when I thought about it more and looked ahead, I realized that I had to be a model for them. I needed to start with myself. And once they saw me doing it, they began to follow and get on track too.”

**Representative Quotes for Theme 3. Focus on messages (Box and noticeboard)**	
**Interview number**	**Sub-theme**
	**3.1. Appreciation and recognition**
5	“Appreciation for the clinical rounds and for some residents for their hard work, and for some nurses for their nursing care of ventilated and comatose patients. Also, the nursing staff started to appreciate each other… and there are many examples, a lot of examples, that happened and were written in the papers. And it makes a very good atmosphere.”
20	“There were some messages exchanged between people, I mean from one shift to another. Like, ‘Physiotherapists are the best’ or ‘We are a great team.’”
	**3.2. Encouragement**
1	“Messages like, ‘We are ready, we are happy, let’s move into this day,’ or, ‘Do it for the patient, for the patient’s family.’”
21	“The main message we often saw on the board was something like, ‘Today is going to be a nice day.’ Simple sentences like that served as gentle reminders that we're here, spending most of our time working and that we need to take care of ourselves too.”
	**3.3. Kindness**
8	“Yes, things like wishing someone good luck for the next 12 h, or little compliments, for example, I told a colleague, ‘You are our sun in the night’.”
14	“It was good because sometimes we’d get little notes like, ‘You did a good job,’ and it was kind of cute. It gave you a boost. It really helped me feel motivated for work.”

Representative Quotes for Theme 4. Local initiatives within the Hello Trial	
**Interview number**	**Sub-theme**
	**4.1. Use supporting tools and resources**
16	“We had boxes inside the ICU, and some people even put candies or small snacks in them for the doctors and nurses.”
26	“When the Hello trial started, I created a WhatsApp group for all the nurses and staff. We began exchanging messages back and forth.”
	**4.2. New rituals and practices**
1	“We created our coffee time at 2 or 3 PM every day. That was a change.”
3	“The morning singing on weekdays was the most extraordinary part. It was something completely different from the usual daily routine in the ICU.”

Representative Quotes for Theme 5. Immediate positive outcomes	
Interview number	Sub-theme
	**5.1. Getting to know one’s colleagues better**
12	“As I said, it really brought about a change, some people opened up and let others see who they truly are. It wasn’t just about being a doctor or a nurse anymore; it was about being a person who comes to work. I think the trial had a really positive impact.”
17	“Getting to know some of the nurses we once thought were introverts - was really surprising. Now, they smile, and we actually talk and have real conversations, all thanks to the Hello Trial.”
	**5.2. Improved respect and appreciation**
8	“I had the impression that my work was valued, I felt more valued as a professional.”
17	“I think the intervention made them more cordial, more respectful to each other, and more appreciative of one another.”
	**5.3. Improved workplace atmosphere**
5	“What I have seen on the ward has really changed. I even advised my colleagues, ‘Why don’t you try this intervention in your ICU?’ I have seen a great change.”
7	“I have the impression that, whereas before people sometimes looked at each other a bit crossly, now almost everyone makes the effort to say hello. They’re not going to have an aperitif together in the evening but at least everyone is making an effort.”
	**5.4. Improved communication**
17	“Communication among staff has changed, it’s improved, especially between nurses and doctors, at least from my perspective.”
26	“Just a simple gesture to ease some tension in the morning can make a big difference. After we started this, nurses began sending text messages to physicians more freely. Before, that kind of communication took a lot of thought, nurses might hesitate, thinking, 'Am I bothering the physician? Is this a bad time?' But once we encouraged open communication and everyone started reaching out to each other, that fear and hesitation began to fade away.”
	**5.5. Improved patient care**
6	“It creates something positive that radiates out. You realize you're not alone, that we’re a team. And yes, that energy really radiates.”
11	“By the way, for the families who came to visit the patients, we also started to be more welcoming.”
	**5.6. Belief in positive results**
4	“I think if we find ourselves in a situation where the working environment is bad, we could take this initiative up and, during the morning huddle, bring both nurses and doctors together to discuss it.”
26	“I think the result will be positive; it has had an impact. This happened at a time when the ICU nurses were about to go on strike, and it helped. As one of the ICU directors, I was trying to ease tensions between patients and nurses, and this initiative actually supported that effort. That’s why I believe the results will be encouraging and positive.”

Representative Quotes for Theme 6. Long-term potential outcomes	
Interview number	Sub-theme
	**6.1. No long-term effects**
23	“In general, I don’t think the trial changed very much. It was good to participate, but maybe it was too short, and I think people forgot about it. If I asked my colleagues now, I’m not sure they would even remember the whole process.”
26	“So, the effects, I still see them right now, but they are going down.”
	**6.2. Partial continuation**
7	“But let’s just say it brought a bit more serenity to the team. The fact that they arrived in the morning, read the notes, and saw the posters, it made a difference. I still haven’t taken the posters down; there’s one on my door and another in the staff room. I’ve left them up.”
20	“We still have a board, but I have to say there have been very few messages since the study ended—maybe only three or four. I don’t know if something happened, but maybe more messages will come.”
	**6.3. Future suggestion & recommendation**
9	“I think it’s a good thing, but it should be sustained. It shouldn’t just be one month. It should be part of a planned curriculum on mental health and support systems in the ICU.”
18	“So, if we want to continue, we’ll probably need to be creative by finding new ways to remind people, ways that trigger different parts of the brain.”

**Theme 1. Understanding of the intervention & initial reactions** (with 6 sub-themes)

Many participants understood the intervention as a way to promote basic politeness (**Sub-theme 1.1. Basic politeness**), strengthen team cohesion (**Sub-theme 1.2. Importance of team cohesion**), and create a positive work environment in the ICU (**Sub-theme 1.3. To create a good atmosphere**). Some also linked the intervention to the prevention of burnout among healthcare professionals (**Sub-theme 1.4. Burnout prevention and its importance,** *Quote: “My understanding was that this intervention was meant to help prevent burnout and to create a more positive, supportive atmosphere.” - Interview 4*). However initial reactions to the intervention were mixed. While several participants expressed enthusiasm (**Sub-theme 1.5. Reaction – Excitement,** *Quote: “Personally, I liked it, I really enjoyed it. It was something new, something we hadn’t done before, so I was very excited. And a lot of us felt the same way.” - Interview 17*), others felt sceptical at the beginning (**Sub-theme 1.6. Reaction – Scepticism,** *Quote: “On the other hand, there were some people who weren’t as interested. They felt it was pointless and considered it a waste of time.” - Interview 20*). Participants observed similar responses among their colleagues within the team.

**Theme 2. Facilitators and barriers to implementation** (with 5 sub-themes)

Participants mentioned several factors that affected the implementation of the intervention, both positively and negatively. One barrier was the doubt among the staff members about the effectiveness of the intervention (**Sub-theme 2.1. Belief that the intervention will not work**, *Quote: “They didn’t even want to try the intervention because they didn’t believe it would help reduce their own burnout in any way.” - Interview 17*). Another challenge was the existing workplace atmosphere in the ICU which, in some cases, made the sudden change in social behaviour feel unnatural or forced for certain participants (**Sub-theme 2.2. The impact of prior atmosphere in the ICU (before trial starts**). In addition, local socio-economic factors were perceived as significant barriers (**Sub-theme 2.3. The impact of socio-economic context**, *Quote: “There are other problems that are organisational, and I don’t think a trial like this can address those kinds of issues. Being friendly with colleagues is valuable, but it doesn’t solve problems that come from outside.” - Interview 19*). Participants also noted that due to the high workload and staff shortage it was challenging to carry out the intervention activities for example to gather everybody for the morning meeting, or to write positive messages regularly (**Sub-theme 2.4. High workload**). Despite these challenges, leadership was identified as a key facilitator of the intervention (**Sub-theme 2.5. Importance of leadership**, *Quote: “My boss played an important role. She was very involved, always reminding us, 'Come on guys, remember to smile!' Every single day. She encouraged us constantly” - Interview 11*).

**Theme 3.** Remembering the messages **(box & noticeboard)** (with 3 sub-themes)

Participants recalled that many of the messages they had written focused on colleague appreciation and recognition. (**Sub-theme 3.1. Appreciation and recognition**, *Quote: “I think I wrote something like, 'You're fantastic nurses, working in the ICU. You're doing a great job, and we're so proud of you. This is your well-deserved recognition.” - Interview 17*). Messages also included words of encouragement (**Sub-theme 3.2. Encouragement**, *Quote: “In the box, they used to write messages to each other, like, 'You did a great job yesterday, I was really impressed by your skills. Keep up the good work.' The notes in the box were more personal.” - Interview 9*). Moreover, many messages showed simple acts of kindness (**Sub-theme 3.3. Kindness**, *Quote: “My experience was really positive. Everyone put special messages in the box for other team members, messages to say congratulations, give thanks, or share positive feedback” - Interview 21*).

**Theme 4. Local initiatives within the Hello Trial** (with 2 sub-themes)

Participants explained that they used various supporting tools and resources during the intervention period. For example, several ICUs created WhatsApp groups to stay connected and some used the message box not only for notes but also to place some candies and small snacks in it. (**Sub-theme 4.1. Use supporting tools and resources**). They also described how the HELLO Trial led to the creation of new team rituals and practices. (**Sub-theme 4.2. New rituals and practices**, *Quote: “Active pause was another change introduced in the ICU, a short break during shifts where team members would do simple exercises, like moving the head, hands, and shoulders. Just a small moment to reset and recharge.” - Interview 2*).

**Theme 5. Immediate positive outcomes** (with 6 sub-themes)

When reflecting on the positive outcomes of the intervention, many participants expressed belief in its positive impact (**Sub-theme 5.6. Belief in positive results**). They mentioned that the trial helped them to get to know their colleagues better (**Sub-theme 5.1. Getting to know one’s colleagues better**). In addition, it improved the respect and appreciation among staff members (**Sub-theme 5.2. Improved respect and appreciation**, *Quote: “I’ve changed my perspective a bit on how I receive reports from my colleagues. I think the intervention helped me appreciate their work more, not just focus on what wasn’t done or which tasks weren’t ticked on the list, but also recognize and value what was accomplished that day.” - Interview 3*), as well as the communication within the team (**Sub-theme 5.4. Improved communication**). Participants noted that the workplace atmosphere became more friendly and welcoming (**Sub-theme 5.3. Improved workplace atmosphere**, *Quote: “I feel there is a before and an after. I’ve started putting things into practice that I wasn’t doing before. These were things I had inside me but wasn’t expressing. What we shared was positive for team spirit and really changed the team dynamic.” - Interview 6*). Finally, some observed positive change in the patient care as a result of the intervention (**Sub-theme 5.5. Improved patient care**, *Quote: “I'm really grateful to have taken part in this project because it improved the way we connect with each other. As I mentioned before, this welcoming atmosphere also helped us work better for our patients.” - Interview 11*).

**Theme 6. Long-term potential outcomes** (with 3 sub-themes)

While many participants believed in the positive impact of the trial, many expressed doubts about its long-term effects (**Sub-theme 6.1. No long-term effects**, *Quote: “When they send notes to each other, it’s a way to share a smile or a joke and break away from the routine, robotic nature of ICU work. But I’m not really sure how long this effect lasts, like with anything, at first it stands out, but after a while, it can become automatic, and the brain starts to block it out.” - Interview 18*). Some participants mentioned that certain components of the intervention are still present in the ICU, for example the posters, and some practices are still being followed. However, they observed that the frequency and intensity of these activities had decreased after the intervention. (**Sub-theme 6.2. Partial continuation**, *Quote: “I created a WhatsApp group for the morning huddle, where everyone could say good morning, share what had happened, and talk about how the night went. It really changed the way we connected, and although we now do it to a lesser extent, it remains part of our routine.” - Interview 26*). Looking ahead, participants suggested that if the trial were to continue, it would be beneficial to introduce new activities and repeat the intervention on a regular basis (**Sub-theme 6.3. Future suggestion & recommendation**, *Quote: “I think it shouldn’t be done just once in a while. It should happen more often, maybe not every month, but more regularly and not just in the ICU.” - Interview 16*).

## Discussion

In this qualitative study, we explored healthcare professionals’ experiences of an intervention aimed at promoting a more supportive work environment and reducing burnout through positive communication. Two complementary sources of qualitative data were analysed: (1) written messages produced as part of the intervention itself, which document how teams appropriated and enacted the proposed tools in practice, and (2) semi-structured interviews conducted several months later, which capture healthcare professionals’ retrospective, subjective experiences of the intervention and its perceived effects. Together, these data provide insight into both the situated practices generated by the intervention and the ways in which participants later interpreted its meaning, benefits, and limitations.

Our interview findings suggest that most participants perceived the intervention as resulting in a positive change in the workplace environment. It also highlights the importance of everyday actions, like the need to value others, acts of kindness and being listened to. Moreover, the findings underline the role of leadership style in supporting and sustaining positive change.

The mid-20th century was a turning point, reflecting a shift in workplace practices that reduced previously more holistic understandings of employees’ needs, which in turn highlighted the importance of cultivating a positive workplace culture that values both performance and employee well-being. Moreover, workplace culture is not a nebulous concept but a dynamic force shaped by various factors, such as societal values (e.g., inclusivity), social dynamics (e.g., employee voice), and organizational practices (e.g., leadership style) [13]. Studies show that a negative workplace culture can hinder professional development, reduce job satisfaction and productivity, all of which can contribute to burnout. While workplace culture can be changed, it requires the entire workplace community to actively contribute [14,17].

While participants’ interpretations show how they understood the purpose and potential of the intervention, their experiences during the implementation phase also shed light to the factors that either supported or hindered its uptake. Our study found that barriers included team scepticism, the existing workplace climate, socio-economic conditions and high workload. Factors that can significantly affect the success of an intervention and should be carefully considered during its design [18]. At the same time, leadership was identified as a key facilitator. Participants explained how encouraging leaders, by setting an example, maintained participation. This insight aligns with prior research on the influence of leadership in shaping workplace culture [6,[19], [20], [21]]. Additionally, several ICUs introduced creative, locally adapted practices (e.g. WhatsApp groups, “active pause” routines, singing together in the morning) which suggests that creativity and flexibility are essential for successful implementation. These local approaches demonstrate how ICU teams adapted the intervention to their specific contexts and integrated it into their everyday working culture. By tailoring the intervention to their needs, participants increased engagement, strengthened team connection, and contributed to the sustainability of the initiative over time.

Analysis of the box and noticeboard messages provides insight into how healthcare professionals appropriated the intervention tools and adapted them to their local contexts. The messages reflect collective norms regarding what counts as appropriate recognition, encouragement, or humour within teams, and how these norms were expressed in written and visual form. In this sense, the message corpus documents practices generated within the intervention framework, rather than participants’ later reflections about the intervention.

While interview accounts and intervention materials point to overlapping themes (e.g. appreciation, kindness, teamwork), they do so from different analytical positions: interviews articulate how participants made sense of the intervention and its effects, whereas written messages capture how these values were performed and circulated during the intervention itself.

Our findings from the interviews suggest that experiences of appreciation and recognition played an important role in making team members feel valued and in boosting morale. The written messages produced during the intervention illustrate how these values were expressed and materially enacted within teams, in line with the intervention’s instructions. Rather than reflecting subjective experience per se, these messages document the forms of recognition that teams collectively chose to express and make visible during the intervention period.

These are key elements of meaningful workplace connection, helping to build mutual respect and emotional support, as also identified in previous studies [6].

Participants described improvements in relationships with colleagues and communication within team members. These outcomes align with existing literature showing that small acts of kindness can enhance psychological safety and team cohesion [22].

Lastly, our study shows the importance of maintaining such interventions over time. Although several participants observed an immediate positive impact of the intervention, they also observed some new behaviours and rituals diminished over time. This highlights the need for regular assessments and targeted efforts, elements also recommended in Greenawald’s STARRS framework (an acronym for Service, Teamwork, Attitude, Reflection, Renewal, and Self-care). The framework was designed to create a supportive work environment, emphasizing that healthcare workers should not only care for patients but also support and encourage one another [22].

As a qualitative component within the HELLO trial, this study has several limitations. First, healthcare professionals were not themselves involved in strategies to improve workplace atmosphere. This may have affected both the implementation process and the sustainability of outcomes over time. In addition, participation was voluntary, which means that those who were more satisfied with the intervention may have been more likely to take part in the interviews. As a result, the perspectives of less satisfied staff may not have been fully captured. In addition, language barriers might have limited how some participants expressed their views. These factors should be considered when interpreting the findings.

The diversity of the interview sample, spanning multiple countries, professional roles, and organisational contexts, allowed for a broad thematic mapping of how the intervention was experienced. However, this heterogeneity also limits the extent to which themes can be interpreted as having uniform meanings across settings. Concepts such as politeness, recognition, leadership, humour, or spirituality are shaped by cultural, social, and professional norms, and may therefore be enacted and experienced differently across contexts. The themes identified in this study should thus be understood as analytical abstractions that capture recurring dimensions of experience, rather than as claims of semantic equivalence or cultural universality.

Finally, whilst the sample size is appropriate for qualitative research and data saturation reached, the number of participants relative to participating ICUs limits external validity. In line with qualitative methodology, the aim was to explore experiences and generate insights rather than to provide generalisable findings.

In conclusion, this qualitative study provides insight into how healthcare professionals experienced the intervention, including perceived benefits, barriers, and factors influencing implementation and sustainability. Participants described improvements in day-to-day interactions and team atmosphere, but also highlighted challenges related to workload, engagement, and maintaining effects over time. These findings can inform future adaptations and implementation strategies for similar low-cost interventions in intensive care units.

## CRediT authorship contribution statement

EA, NKB and MC designed this cluster RCT to evaluate the effectiveness of the HELLO bundle. CB designed, led, analysed, and/or advised the pre-post study that preceded this RCT. AB conducted the interviews in English. AB and NKB performed the thematic analysis and drafted the manuscript. All authors revised the manuscript and approved the final version.

## Consent for publication

Participants consented to the sharing of de-identified data and results. Model consent forms will be freely available with other study documents.

## Ethics approval and consent to participate

This study has been approved by the appropriate IRB in each country and validated by each research department at each hospital. Self-administered written informed consent is obtained from all participants.

## Declaration of Generative AI and AI-assisted technologies in the writing process

During manuscript preparation, the authors used ChatGPT (OpenAI) to transcribe text from images (box and noticeboard messages) and DeepL to translate the text into English. All transcriptions and translations were verified against the original images and edited as needed. The authors take full responsibility for the final content.

## Funding

This research study and program are supported by the European Society of Intensive Care Medicine.

## Availability of data and material

Data will not be shared.

## Declaration of competing interest

EA has received fees for lectures from Gilead, Sanofi, Alexion and Pfizer. His institution has received research support from Mindray and Pfizer. All other authors report no conflict of interest.
